# Impact of the 2015 Dutch Long‐Term Care Reform on Nursing Home Use and Access for People With Dementia

**DOI:** 10.1111/jgs.70301

**Published:** 2026-01-22

**Authors:** Joost D. Wammes, Bram Wouterse, Terrence E. Murphy, Janet L. MacNeil Vroomen

**Affiliations:** ^1^ Department of Internal Medicine, Section of Geriatric Medicine Amsterdam University Medical Center, University of Amsterdam Amsterdam the Netherlands; ^2^ Ageing and Later Life, Amsterdam Public Health Research Institute Amsterdam the Netherlands; ^3^ IQ Health, Radboud University Medical Center Nijmegen the Netherlands; ^4^ Department of Public Health Sciences Penn State College of Medicine Hershey Pennsylvania USA; ^5^ Section of Geriatrics, Department of Internal Medicine Yale School of Medicine New Haven Connecticut USA

**Keywords:** dementia, health policy, long‐term care, nursing homes

## Abstract

**Background:**

In 2015, the Netherlands implemented long‐term care (LTC) reforms to promote aging‐in‐place, potentially impacting nursing home (NH) access for older individuals with dementia. This study examines how the reform affected NH admission rates and waiting list prevalence for this population.

**Methods:**

We performed interrupted time series analyses to evaluate trends in NH admissions (2011–2019, Statistics Netherlands) and waiting list prevalence (2013–2018, National Healthcare Institute) before and after the 2015 LTC reform. Incidence rate ratios (IRR) were calculated for monthly NH admission rates and waiting list prevalence.

**Results:**

Among 270,706 older people with dementia, the reform was negatively associated with NH admission rates (IRR 0.610 [0.547–0.681]), halting the pre‐reform decline and stabilizing the post‐reform trend (IRR 1.001 [0.999–1.002]). The reform was positively associated with NH waiting list prevalence (IRR 1.159 [1.048–1.282]).

**Conclusion:**

Among older Dutch people with dementia, the 2015 Dutch LTC reform was associated with fewer NH admissions and longer waiting lists. While stabilization of the NH admissions may reflect prioritization of persons with dementia within stricter eligibility criteria, the concurrent rise in waiting list prevalence suggests that institutional capacity did not keep pace with persistent need. As a result, many older people with dementia remain longer in the community, raising concerns regarding their health and safety as well as the burden on their informal caregivers.

## Summary

- Key points
  ◦ The 2015 Dutch long‐term care reform promoted aging‐in‐place by limiting nursing home access to only those with severe care needs.
  ◦ The reform was associated with declines in nursing home admissions and increases in waiting lists among older adults with dementia.
  ◦ These outcomes reveal an unintended consequence of aging‐in‐place policies, that is, that of prolonged residence in a community of older persons with dementia.
- Why does this paper matter?
  ◦ This study provides population‐level evidence on the unintended effects of long‐term care reforms designed to promote aging‐in‐place. While such policies aim to support autonomy and reduce use of institutional care, the findings show reduced admissions and longer waiting lists among people with dementia. These results highlight the need for long‐term care strategies that ensure timely access to appropriate care for those who need high levels of support.

## 1 Introduction

The Netherlands spends more than twice the per capita average on long‐term care (LTC) in Organization for Economic Co‐operation and Development (OECD) countries [1]. Over 80% of these expenditures go toward care in nursing homes (NH), rather than community‐based care [1, 2]. To address the rising costs of LTC, the Netherlands introduced a major LTC reform in 2015 that primarily focused on supporting older adults to age‐in‐place by limiting NH admission to those requiring 24/7 care or supervision [3, 4]. Dementia often requires a high level of care that places a significant burden on informal caregivers [5, 6, 7]. Therefore, reducing NH admissions and increasing community living time could disproportionately impact individuals with dementia and their informal caregivers.

Previous population‐level research found that persons with dementia had increased risk of hospital deaths after the LTC reforms, but there was no change in the rate of death at home [8]. One possible explanation for this may be a decrease in the utilization of NH by this subpopulation. Even though persons with dementia make up around half of the total Dutch population in NH [5], it is currently unclear how the 2015 reform influenced their utilization of NH.

In this study we pose two related questions regarding older Dutch persons using dementia related care. The first is whether the 2015 Dutch LTC reform was associated with a decrease in the rate of NH admission. The second question is whether the reform was associated with an increase in the national waiting list prevalence for NH placement. Beyond the Dutch context, these questions are directly relevant for US policy and practice: understanding how reforms influence aging‐in‐place and long‐term care utilization can inform efforts to optimize dementia care while supporting informal caregivers, consistent with themes highlighted in the American Geriatrics Society's *Strategic Framework for a National Plan on Aging* [9]. This study provides the first longitudinal insights regarding the association of the 2015 Dutch LTC reform with NH utilization by older Dutch persons with dementia.

## 2 Methods

### 2.1 Study Design

An interrupted time series (ITS) was used to evaluate whether the Dutch national LTC reform of 2015 was associated with both immediate and persistent changes in the monthly rate of NH admission and with the waiting list prevalence of persons with dementia waiting for NH placement [10]. This study followed the RECORD statement for study design and reporting [11].

### 2.2 Study Data

For the analyses of NH admissions, Statistics Netherlands provided person level episodic NH data for the years 2009 to 2018, covering the total population of Dutch adults 65 years and older. Aside from an income‐commensurate co‐payment, care in NH is fully financed under the National Long‐term Care Scheme (in Dutch Wet Langdurige Zorg) [4]. NH eligibility is based on an independent assessment of social and medical needs conducted by the Center for Needs Assessment (Centrum Indicatiestelling Zorg, CIZ) that results in a package score which determines the type and amount of care that can be received; higher package scores (hereafter care score) authorize higher levels of care. Care scores 5–8 correspond to eligibility for NH admission. The care scores that make a person with dementia eligible for admission to a Dutch NH are ZZP 5 (protective living with intensive dementia care), and care scores ZZP 6–8 (protective living with intensive care and nursing). To discern “true admissions” from administrative artifacts, an admission counts as new admission if there are at least 30 days between the new admission and the discharge of the prior admission [8]. The person level NH utilization data were linked to municipality registration records by an anonymized citizen service number with nearly perfect matching (> 99%). Municipality data included individual characteristics like age, sex, and mortality. For the ITS analysis, we collapsed person level data into population level monthly rates of NH admission.

For the analysis of NH waiting lists, the National Healthcare Institute provided national monthly aggregated waiting list data for people on the waiting list with care score ZZP 5 (protective living with intensive dementia care). Following CIZ assessment and care score assignment, persons deemed eligible for NH admission can be placed on a waiting list for either immediate placement in any available facility or for placement in their preferred NH at a later date. Once on the waiting list, individuals remain in their current living situation (typically at home with community‐based support) until a NH place becomes available. It was not possible to identify people with dementia on NH waiting lists for care scores ZZP 6–8 as this data is not available. The data consisted of the monthly count of persons on the waiting list for the period February 2013 to April 2018. This count includes both persons that are waiting for direct NH placement and those waiting for a placement at their NH of choice.

### 2.3 Primary Outcomes

The monthly rate of NH admission was calculated as the ratio of total monthly count of new NH admissions of persons with dementia over the total monthly weighted population aged 65 years and older that was eligible for NH admission. The monthly NH waiting list prevalence of persons with dementia was calculated as the ratio of the total monthly count of persons with dementia on the NH waiting list over the total monthly weighted population aged 65 years and older.

### 2.4 Statistical Analysis

Descriptive statistics of the NH residents admitted with dementia, the NH person‐admissions, and the NH waiting list prevalence were presented for the pre‐ and post‐reform periods. The yearly unadjusted mean of monthly count of NH admission, admission rate, waiting list count, and waiting list prevalence were also calculated.

#### 2.4.1 Interrupted Time Series Analysis (ITS)

For the two rate outcomes (rate of NH admission and NH waiting list prevalence), a negative binomial regression analysis was used to calculate adjusted incidence risk ratios with a log link, per the following ITS model:

$$\mathrm{Log}\;Y_{t}=\beta_{ο}+\beta_{1}\text{time}+\beta_{2}{\text{reform}}_{t}+\beta_{3}{\text{reform}}_{t}*\text{time}+\beta_{4}{\text{seasonality}}_{t}$$

where β_0_ is the outcome at time 0, β_1_ represents the association of time over the pre‐reform period, that is, pre‐reform trend, and β_2_ is the association of reform, that is, the average reform effect. The average effect of reform is equivalent to the ratio of the least square mean estimate from the post‐reform period over that of the pre‐reform period. The association of the interaction term β_3_ represents the change in trend in the post‐reform period relative to that of the pre‐reform period, that is, the change in trend. The regression model was adjusted for seasonality (β_4_) by the inclusion of monthly radians based on a cosine function with a period of 12 months [12]. For the waiting list prevalence outcome, because the inclusion of the cosine function was not significant and did not improve model fit, we did not adjust for seasonality. The immediate changes directly following the reform (level change) were calculated as the ratios of the adjusted rates from January 2015 over those of December 2014 and were presented as percentages. The monthly trend of each outcome in the post‐reform period (post‐reform trend) was calculated as the linear combination of the pre‐reform trend and the estimated association of the interaction. All model coefficients were estimated using robust standard errors and reported as incident rate ratios (IRR), which are the exponentiation of the regression coefficients and their corresponding 95% CIs. The IRR represent the proportional change in the outcome corresponding to incremental change in the relevant explanatory variable. To facilitate interpretation, the two rate outcomes were scaled to events per 100,000 older adults. All analyses were conducted using Stata 16.0 with statistical significance defined as a 2‐tailed *p*‐value < 0.05.

### 2.5 Sensitivity Analysis

For the interrupted time series analyses, we conducted structural break tests for both known (Chow test) [13] and unknown breaks (Supremum Wald test with symmetric trimming of 15%) [14]. The known break test included the exclusion from NH admission of lower care score persons (ZZP 1–3) that started in 2013, and which eliminated access to LTC that was previously available to persons with lower care scores. In addition, a one‐year lagged outcome term was added to evaluate whether there were potentially maturing effects of the development of care services following the reform. The results and interpretation of the sensitivity analyses are in Supporting Information.

## 3 Results

### 3.1 Study Characteristics

Table 1 compares characteristics such as age, sex, and care score between LTC residents with dementia admitted in the pre‐ and post‐reform periods, respectively. The average unadjusted mean of monthly count of NH admission and the monthly rate of NH admission were significantly lower in the post‐reform period. In contrast, the average unadjusted mean monthly count of persons waiting for NH admission and waiting list prevalence were significantly higher in the post‐reform period. Year‐specific data on mean monthly count of NH admissions, monthly rates of NH admission, monthly counts of the NH waiting list, and monthly NH waiting list prevalence can be found in the Supporting Information.

**TABLE 1:** Characteristics of Nursing Home (NH) residents, NH person‐admissions, and NH waiting lists for Older Dutch Persons with Dementia during Pre‐ and Post‐Reform Periods.

Characteristics	Pre‐reform	Post‐reform	*p*
Dementia nursing home residents (*N* = 270,706)	*N* = 130,396	*N* = 140,310	
Mean Age (IQR)	85.8 (81.5–90.8)	85.3 (80.9–90.2)	< 0.001*
Sex (%)			< 0.001**
Female	86,598 (66.41%)	89.484 (63.78%)	
Male	43,799 (33.59%)	50,826 (36.22%)	
Care score at admission^†^			< 0.001**
ZZP 5	100,148 (76.80%)	132,137 (94.18%)	
ZZP 6	323 (0.25%)	413 (0.29%)	
ZZP 7	29,828 (22.87)	7691 (5.48%)	
ZZP 8	97 (0.07%)	69 (0.05%)	
Nursing home admissions	*N* = 130,396	*N* = 140,310	
Mean monthly count of nursing home admissions (SD)	2716 (421.7)	2338.5 (211.7)	< 0.001*
Mean monthly nursing home admission rate per 100,000 adults aged ≥ 65 (SD)	59.6 (10.6)	47.1 (4.0)	< 0.001*
Nursing home waiting list (*N* = 281,032)	*N* = 86,181	*N* = 194,851	
Mean monthly waiting list prevalence count (SD)	3747 (79.7)	4871 (73.1)	< 0.001*
Mean monthly waiting list prevalence per 100,000 adults aged ≥ 65 (SD)	80.0 (18.5)	99.5 (13.1)	< 0.001*

Abbreviations: 95% CI, 95% confidence interval; IQR, inter quartile range, SD, standard deviation.

* Independent *t*‐test.

** Chi‐square.

^†^ Care scores (ZZP packages) range from 1 to 8 and indicate increasing levels of care dependency; scores 5–8 determine eligibility for NH admission. ZZP 5 corresponds to protective living with intensive dementia care, whereas ZZP 6–8 correspond to protective living with intensive care and nursing.

A surprising finding was that in the post‐reform period, proportions of care score allocation (where higher scores indicate higher intensity care) for NH admission shifted from higher care scores of ZZP 6–8 (protective living with intensive care and nursing) toward the lower care score of ZZP 5 (protective living with intensive dementia care); this was an increase of 17% of those with care score ZZP 5. In the post‐reform period over 94% of persons with dementia were admitted with a care score of ZZP 5. This was counter‐intuitive because care score ZZP 7 is typical for persons with dementia that have severe behavioral problems. However, the proportion of NH admissions with care score ZZP 7 decreased from 23% to 5% in the post‐reform period. It remains unclear what caused the shift toward the lower care scores.

### 3.2 Interrupted Time Series of Monthly Rate of Nursing Home Admission for Persons With Dementia

**FIGURE 1:**
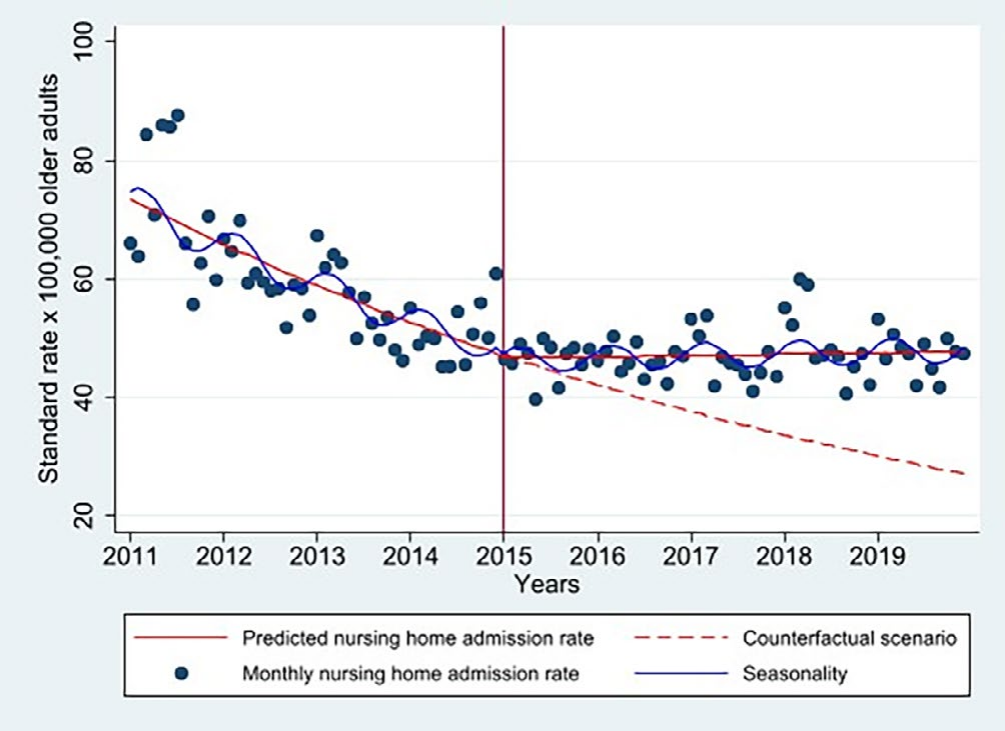
Interrupted time series of monthly rate of admission of older Dutch persons with dementia to nursing homes (*N* = 270,706). The blue dots represent the observed monthly rate of nursing home admissions per 100,000 older adults. The blue curve illustrates the within‐year seasonal pattern, as estimated using a cosine function. The red solid lines show the predicted monthly admission rate based on the adjusted negative binomial regression model. The red dashed line depicts the counterfactual scenario, estimating what the monthly admission rate might have been had the reform not occurred (negative binomial regression model). In the post‐reform period, the predicted admission rate exceeds the counterfactual estimate, suggesting that admissions might have been lower if the reform had not taken place.

Figure 1 and Table 2 show that in the pre‐reform period the monthly NH admission rate exhibited a significantly decreasing trend with an IRR of 0.991 (95% CI 0.988–0.993, *p* < 0.001), equivalent to an expected decrease of 0.9% per month. The implementation of the reform resulted in a negative step change of −1.4%. This was followed by a positive change in trend with an IRR of 1.009 (95% CI 1.007–1.012, *p* < 0.001), which resulted in a flat post‐reform trend with an IRR of 1.001 (95% CI 0.999–1.002, *p* = 0.204), that is, a net zero slope over the post‐reform period. Overall, there was a negative average effect of reform with an IRR of 0.610 (95% CI 0.547–0.681, *p* < 0.001); this corresponds to the ratio of the least square mean point estimate from the post‐reform period (41.99 admissions per 100,000 older adults) over that of the pre‐reform period (68.85 admissions per 100,000 older adults).

**TABLE 2:** Adjusted incident risk ratio (IRR) of nursing home admission rate for persons with dementia from interrupted time series analysis (*N* = 270,706).

	Incident rate ratio (95% CI), *p*
Pre‐reform trend (β_1_)	0.991 (0.988–0.993), *p* < 0.001
Change in Trend (β_3_)	1.009 (1.007–1.012), *p* < 0.001
Post‐reform trend (β_1_ + β_3_)	1.001 (0.999–1.002), *p* = 0.204
Average reform effect (β_2_)	0.610 (0.547–0.681), *p* < 0.001

*Note*: An IRR quantifies the multiplicative rate of change in the outcome per unit time. An IRR < 1.0 indicates a decreasing rate, IRR = 1.0 indicates no change, and IRR > 1.0 indicates an increasing rate. The pre‐reform trend IRR of 0.991 indicates that nursing home admission rates were declining by approximately 0.9% per month before the reform (1–0.991 = 0.009 or 0.9% decrease per month). The average reform effect shows that the rate of admission across the entire post‐reform period was, on average, 39% lower than the rate of admission across the entire pre‐reform period (1–0.610 = 0.39).

Abbreviations: 95% CI, 95% confidence interval; IRR, incident rate ratio.

### 3.3 Interrupted Time Series of Nursing Home Waiting List Prevalence for Older Dutch Persons With Dementia

**FIGURE 2:**
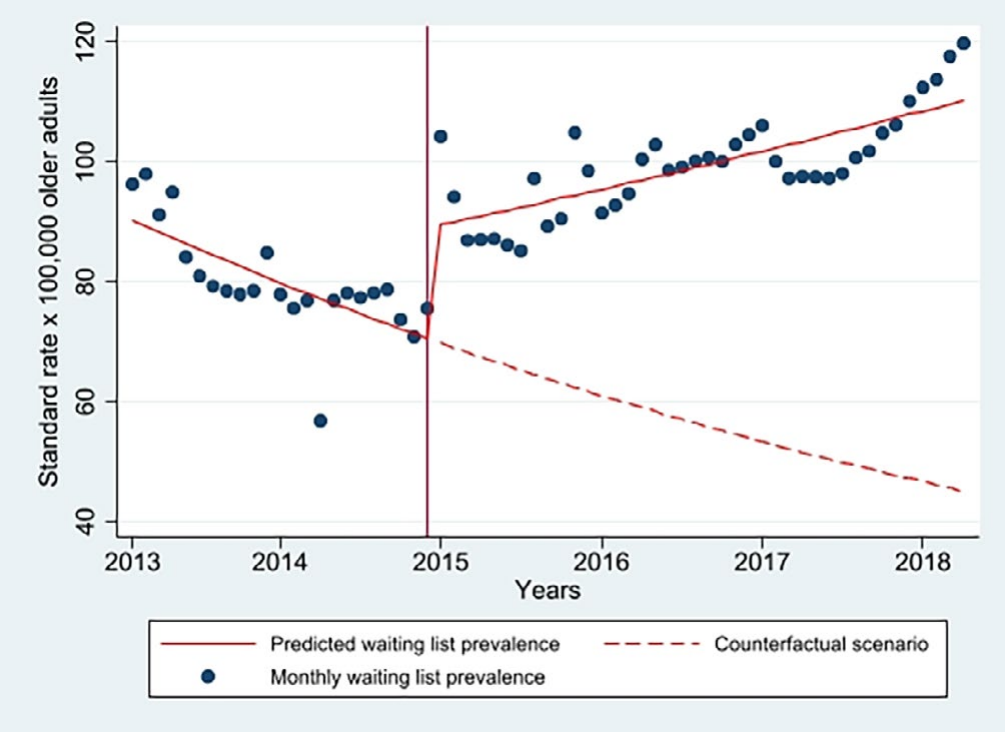
Interrupted time series on nursing home waiting list prevalence for older Dutch persons with dementia (*N* = 281,032). The blue dots represent the observed monthly waiting list prevalence per 100,000 older adults. The red solid lines show the predicted monthly waiting list prevalence based on the adjusted negative binomial regression model. The red dashed line depicts the counterfactual scenario, estimating what the monthly waiting list prevalence might have been had the reform not occurred (negative binomial regression model). In the post‐reform period, the predicted waiting list prevalence exceeds the counterfactual estimate, suggesting that the waiting list prevalence might have been lower if the reform had not taken place. Twelve‐month seasonality is not presented because its inclusion did not improve model fit.

Figure 2 and Table 3 show that in the pre‐reform period, the monthly NH waiting list prevalence of older Dutch persons with dementia exhibited a decreasing trend with an IRR of 0.989 (95% CI 0.985–0.993, *p* < 0.001). This is equivalent to an expected decrease of 1.1% per month. The reform implementation resulted in an immediate positive step change of 26.8% that was followed by a positive change in trend with an IRR of 1.016 (95% CI 1.012–1.021, *p* < 0.001). That in turn resulted in a positive post‐reform trend with an IRR of 1.005 (95% CI 1.004–1.007, *p* < 0.001), which is equivalent to an increase of 0.5% per month. Overall, there was a positive average effect of reform with an IRR of 1.159 (95% CI 1.048–1.282, *p* = 0.004); this corresponds to the ratio of the least square mean rate in the post‐reform period (103.1 waiting per 100,000 older adults) over that in the pre‐reform period (88.9 waiting per 100,000 older adults).

**TABLE 3:** Adjusted incidence rate ratio (IRR) of nursing home waiting list prevalence of older Dutch persons with dementia from interrupted time series analysis (*N* = 281,032).

	IRR (95% CI), *p*
Pre‐reform trend (β_1_)	0.989 (0.985–0.993), *p* < 0.001
Change in Trend (β_3_)	1.016 (1.012–1.021), *p* < 0.001
Post‐reform trend (β_1_ + β_3_)	1.005 (1.004–1.007), *p* < 0.001
Average reform effect (β_2_)	1.159 (1.048–1.282), *p* = 0.004

*Note*: The change in trend (IRR of 1.016) indicates that the slope of the waiting list prevalence across the entire pre‐reform period increased, on average, by approximately 1.6% per month relative to the slope over the entire pre‐reform period. The slope in the post‐reform period is the linear combination of the pre‐reform slope and this change term.

Abbreviation: IRR, incident rate ratio.

## 4 Discussion

In this longitudinal study of older Dutch persons with dementia, the Dutch 2015 LTC reform was associated with fewer NH admissions and increased waiting list prevalence. While the rate of admission was decreasing over the pre‐reform period, the initiation of the reform appears to have halted this decline and was followed by a rate that was flat over the post‐reform period. The implementation of the reform was also associated with an immediate and sustained increase in the NH waiting list prevalence. The latter represented a reversal of the decreasing trend in the NH waiting list prevalence observed in the pre‐reform period.

So is the reform all bad news for older Dutch persons with dementia who need LTC? It would be easy to draw that conclusion without some important context from a similar analysis of the complete cohort of older Dutch persons. MacNeil Vroomen et al. [15] evaluated the effect of the 2015 Dutch LTC reform on NH utilization across the complete older Dutch population. Notably, the full cohort study also documented a substantial increase in the proportion of admissions assigned to care score ZZP 5 (the lower intensity dementia‐specific care package) similar to the shift observed in the current study [15]. A comparison of the effects of the 2015 national reform on the complete cohort with its effects on the subgroup of older Dutch persons with dementia offers two pieces of evidence suggesting that persons with dementia were prioritized for NH admission in the post‐reform period. These pieces of evidence represent the short and long term changes in rate of NH admissions assessed during the post‐reform period. The short term observation is that in the full cohort the rate of NH admission experienced a large negative step change of 19% immediately after initiation of the reform [15]. In contrast the subgroup of persons with dementia underwent a much smaller negative step change of 1.4%. The longer term observation compares the changes in average trend from the pre‐ to post‐reform periods in each of the two cohorts; this change is represented by the interaction term in the model. In the full cohort the trend in rate of NH admission changed by 1.004 (*p* = 0.017) whereas a change of 1.009 (*p* < 0.001) was observed in the subgroup with dementia. Because of the restricted admission criteria of the reform, both the full cohort and subgroup of persons with dementia exhibited an overall decrease in the rate of NH admission during the post‐reform period. But the finer grained comparison of their temporal dynamics suggests that older persons with dementia were prioritized for NH admission in the post‐reform period. This is consistent with findings from Japan showing that during governmental intervention, due to their complex needs, persons with dementia were prioritized for NH placement [16].

Nonetheless, our findings regarding NH waiting list prevalence suggest that in the post‐reform period NH admissions are being delayed for older Dutch persons with dementia. The increase in the waiting list prevalence of 28% immediately following the reform, which was followed by an estimated 5‐year increase of approximately 35%, shows that many older Dutch persons with dementia who are eligible for NH placement continue to reside at home. This delayed NH admission can be particularly harmful for the dementia population; Bär et al. [17] found that in this population a delay of a single month is associated with a 3.1% increased probability of hospitalization. This corroborates earlier research reporting that the 2015 Dutch reform was associated with increased hospital utilization [18]. In addition, the reform was associated with higher mortality among older Dutch persons with dementia in hospital relative to mortality in NH [8]. Extending time at home may cause negative consequences for persons with dementia as well as for their informal caregivers, who might be unreasonably burdened with these increasingly complex caregiving situations [19]. A systematic review of informal caregivers of people with dementia awaiting NH placement shows they experience a persistent need for emotional and informational support [20]. Effective interventions should address decision‐making, emotional challenges, and collaboration with healthcare providers, based on the identified needs of patients and their caregivers [20].

This study has several limitations that warrant mention. The individual level NH utilization data included a small number (< 3%) of persons with more than one admission. It was not possible to determine if these were actual admissions or administrative artifacts. Furthermore, the NH admission rate data showed indications of non‐stationarity, the latter a prerequisite for time series analysis. In addition, the LTC waiting list data exhibited sizable variability over the study period, especially in the pre‐reform period, that was not well accounted for in the model. The aggregated nature of the data precluded identification of specific factors driving this variability, though it likely reflects a combination of fluctuations in NH bed capacity and turnover, changes in referral patterns, and system‐level adjustments in anticipation of the 2015 reform. An important limitation is the absence of data on NH bed capacity and occupancy rates during the study period. The concurrent decline in admissions and rise in waiting lists suggests supply‐side constraints, potentially reflecting NH closures, unit downsizing, or increased length of stay among residents with higher care needs but we cannot definitively assess these mechanisms without capacity data. Future research examining the relationship between policy‐driven changes in admission rates, facility capacity adjustments, and occupancy dynamics would provide valuable insights into the full impact of long‐term care reforms. Another limitation was that for the NH waiting list prevalence outcome, only data of persons with care score ZZP 5 were included in the time series analysis. While our admission data from Statistics Netherlands allowed identification of persons with dementia across all care scores (ZZP 5–8), the aggregated waiting list data from the National Healthcare Institute only provided separate counts for persons with dementia waiting for care score ZZP 5. Due to the low overall proportion of admissions with care scores ZZP 6–8 (less than 6% post‐reform), we believe this limitation had minimal influence on our findings.

Our model findings regarding rates of NH admission suggest that since implementation of the Dutch 2015 LTC reform, admissions of older Dutch persons with dementia decreased by around 39% relative to the pre‐reform period, which was characterized by great variability. This result agrees with the increase in waiting list prevalence, which suggests that NH admissions of older persons with dementia were, on average, delayed during the post‐reform period. One constructive effect of the Dutch 2015 LTC reform was that the rate of NH admission of older Dutch persons with dementia stabilized from its previous state of volatility, which can be regarded as a positive outcome for planning purposes. However, the concurrent rise in waiting list prevalence indicates that this stabilization was achieved under conditions of constrained capacity. While stricter eligibility criteria likely prioritized persons with dementia for admission, the supply of NH places did not keep pace with demand, leaving many to remain at home despite eligibility.

Taken together, the reform stabilized NH admission rates for persons with dementia but did so under constrained capacity, as evidenced by rising waiting lists. These findings emphasize the need to balance policies promoting aging‐in‐place with adequate long‐term care capacity, consistent with the American Geriatrics Society's *Strategic Framework for a National Plan on Aging* and carry direct implications for shaping US dementia care and long‐term services and supports [9].

## Author Contributions

**Joost D. Wammes:** study concept, study design, data management, statistical analysis, drafting of manuscript. **Bram Wouterse:** interpretation of results, drafting of manuscript, and critical revision of manuscript. **Terrence E. Murphy:** study concept, study design, interpretation of results, drafting of manuscript, manuscript review, supervision. **Janet L. MacNeil Vroomen:** study concept, study design, interpretation of results, drafting of manuscript, manuscript review, supervision.

## Funding

This work was supported by Alzheimer Nederland (WE.15‐2021‐11, WE.06‐2021‐04), Amsterdam Public Health Research Institute (2024012), National Center for Advancing Translational Sciences, National Institutes of Health (UL1 TR002014), Yale Claude D. Pepper Older Americans Independence Center (P30AG021342), ZonMw (09150172110097).

## Disclosure

The sponsors had no role in the design, methods, subject recruitment, data collection, analysis, or preparation of the manuscript.

## Conflicts of Interest

The authors declare no conflicts of interest.

## Supporting information


**Data S1:** jgs70301‐sup‐0001‐Supinfo.pdf.
