# Supplementary material for: Impact of the 2015 Dutch Long‐Term Care Reform on Nursing Home Use and Access for People With Dementia
Source: J Am Geriatr Soc. 2026 Jan 22;74(3):722–8. doi: 10.1111/jgs.70301 (PMC12968352; doi:10.1111/jgs.70301)
Supplement: Supplementary file 1 — Data S1: jgs70301‐sup‐0001‐Supinfo.pdf. [file JGS-74-722-s001.pdf]

Supplementary Material S1

1. Sensitivity analysis

1.1 Structural break testing long-term care (LTC) admission rate data

Table A1 shows that the structural break test for known breaks identifies January 2013 and January 2015 as significant breaks in the time series data. In addition, table A1 shows that the break test for unknown break identifies July 2013 as significant break in the time series data with the highest Wald statistic.

Table A1. Structural break test for known and unknown breaks.

	**Wald statistic, P - value**
**Known brake date**	
January 2013	65.48 < 0.001
January 2015	110.72 < 0.001
**Unknown break date**	
July 2013	161.96 < 0.001

1.2 One year lagged time series LTC admission rate

Table A2 shows the adjusted incident risk ratio (IRR) of the LTC admission rate from the one year lagged interrupted time series analysis, omitting 2015 data. The parameter estimates are comparable with the estimates of the main interrupted time analysis (full data model), and 95% CI overlap (see manuscript table two). This could suggest that there was minimal maturation of health care services in the first year after the reform.

Table A2. Adjusted IRR of LTC admission rate from the one year lagged interrupted time series analysis.

	**IRR (95% CI), P-value**
**Pre-reform slope**	0.991 (0.988 - 0.994) < 0.001
**Slope change**	1.010 (1.006 – 1.013) < 0.001
**Post-reform slope**	0.001 (-0.001 - 0.002) = 0.283
**Average reform effect**	0.614 (0.548 – 0.689) < 0.001

1.3 Structural break testing waiting list data

Table A3 shows that the break test for unknown break identifies January 2015 as significant break in the time series data with the highest Wald statistic. There was no break testing required for known breaks, as data on waiting list ranged from 2013-2018. In this period there were no other relevant reforms identified in literature.

Table A3. Structural break test for known break.

	**Wald statistic, P-value**
**Unknown break date**	
January 2015	65.5, < 0.001

1.4 One year lagged time series waiting list rate

Table A4 shows the adjusted incident risk ratio (IRR) of the waiting list rate from the one year lagged interrupted time series analysis, omitting 2015 data. The parameter estimates are comparable with the estimates of the main interrupted time analysis (full data model), and 95% CI overlap (see manuscript table four). This could suggest that there was minimal maturation of health care services in the first year after the reform.

Table A4. Adjusted IRR of waiting list rate from the one year lagged interrupted time series analysis.

	**IRR (95% CI), P-value**
**Pre-reform slope**	0.988 (0.985 - 0.993) $p = 0.001$
**Slope change**	1.017 (1.012 - 1.021) $p < 0.001$
**Post-reform slope**	1.006 (1.004 - 1.008) $p < 0.001$
**Average reform effect**	1.206 (1.084 - 1.342) $p < 0.001$

2. Unadjusted year data

2.1 Unadjusted yearly data of LTC facility admissions and LTC facility admission rate.

Table A5. Per year unadjusted mean monthly LTC facility admission and mean monthly LTC facility admission rate (N=270,706).

	**Mean monthly LTC admission count (SD)**	**Mean monthly rate of LTC admission* (SD)**
**2011**	3,151 (493)	71.6 (11.3)
**2012**	2,716 (211)	60.0 (5.0)
**2013**	2,586 (305)	55.8 (6.9)
**2014**	2,413 (231)	51.0 (4.8)
**2015†**	2,232 (147)	46.5 (3.1)
**2016†**	2,256 (112)	46.3 (2.3)
**2017†**	2,299 (197)	46.5 (4.2)
**2018†**	2,473 (298)	49.2 (6.2)
**2019†**	2,430 (166)	47.4 (3.3)

*(Monthly long-term care facility admissions count/ monthly standardized total population 65 years and older) x 100,000.

† Post-reform years

2.2 Unadjusted yearly data of LTC facility waiting list count, and waiting list rate

Table A6. Per year unadjusted mean monthly LTC facility waiting list count and mean monthly waiting list rate (February 2013 - April 2018, N=281,032).

	**Mean monthly waiting list count (SD)**	**Mean monthly waiting list rate* (SD)**
**2013**	3980 (340)	85.9 (7.8)
**2014**	3533 (288)	74.7 (6.1)
**2015†**	4451 (345)	92.6 (7.1)
**2016†**	4831 (216)	99.0 (4.1)
**2017†**	5023 (231)	101.5 (4.4)
**2018†**	5795 (180)	115.9 (3.4)

*(Monthly waiting list count / monthly standardized total population 65 years and older) x 100,000.

† Post-reform years

The RECORD statement – checklist of items, extended from the STROBE statement, that should be reported in observational studies using routinely collected health data.[1]

	Item No.	STROBE items	Location in manuscript where items are reported	RECORD items	Location in manuscript where items are reported
**Title and abstract**					
	1	(a) Indicate the study's design with a commonly used term in the title or the abstract (b) Provide in the abstract an informative and balanced summary of what was done and what was found	1a. Title<br>1b. Abstract	RECORD 1.1: The type of data used should be specified in the title or abstract. When possible, the name of the databases used should be included.<br><br>RECORD 1.2: If applicable, the geographic region and timeframe within which the study took place should be reported in the title or abstract.<br><br>RECORD 1.3: If linkage between databases was conducted for the study, this should be clearly stated in the title or abstract.	Title and abstract<br><br>Title and abstract<br><br>Abstract
**Introduction**					
Background rationale	2	Explain the scientific background and rationale for the investigation being reported	Abstract and introduction		Abstract and introduction
Objectives	3	State specific objectives, including any prespecified hypotheses	Last paragraph of introduction		Complete
**Methods**					

Study Design	4	Present key elements of study design early in the paper	Study design section		Design section
Setting	5	Describe the setting, locations, and relevant dates, including periods of recruitment, exposure, follow-up, and data collection	Methods		Methods
Participants	6	*(a) Cohort study* - Give the eligibility criteria, and the sources and methods of selection of participants. Describe methods of follow-up<br>*Case-control study* - Give the eligibility criteria, and the sources and methods of case ascertainment and control selection. Give the rationale for the choice of cases and controls<br>*Cross-sectional study* - Give the eligibility criteria, and the sources and methods of selection of participants<br><br>*(b) Cohort study* - For matched studies, give matching criteria and number of exposed and unexposed<br>*Case-control study* - For matched studies, give matching criteria	Methods section	RECORD 6.1: The methods of study population selection (such as codes or algorithms used to identify subjects) should be listed in detail. If this is not possible, an explanation should be provided.<br><br>RECORD 6.2: Any validation studies of the codes or algorithms used to select the population should be referenced. If validation was conducted for this study and not published elsewhere, detailed methods and results should be provided.<br><br>RECORD 6.3: If the study involved linkage of databases, consider use of a flow diagram or other graphical display to demonstrate the data linkage process, including the number of individuals with linked data at each stage.	Study data section<br><br>Study data section<br><br>Methods

		and the number of controls per case			
Variables	7	Clearly define all outcomes, exposures, predictors, potential confounders, and effect modifiers. Give diagnostic criteria, if applicable.	Methods	RECORD 7.1: A complete list of codes and algorithms used to classify exposures, outcomes, confounders, and effect modifiers should be provided. If these cannot be reported, an explanation should be provided.	Methods
Data sources/ measurement	8	For each variable of interest, give sources of data and details of methods of assessment (measurement). Describe comparability of assessment methods if there is more than one group	Methods		
Bias	9	Describe any efforts to address potential sources of bias	Methods		Methods and limitations section
Study size	10	Explain how the study size was arrived at	Methods		Methods
Quantitative variables	11	Explain how quantitative variables were handled in the analyses. If applicable, describe which groupings were chosen, and why	Methods		Methods
Statistical methods	12	(a) Describe all statistical methods, including those used to control for confounding<br>(b) Describe any methods used to examine subgroups and interactions	Methods-statistical analysis		

		(c) Explain how missing data were addressed<br>(d) *Cohort study* - If applicable, explain how loss to follow-up was addressed<br>*Case-control study* - If applicable, explain how matching of cases and controls was addressed<br>*Cross-sectional study* - If applicable, describe analytical methods taking account of sampling strategy<br>(e) Describe any sensitivity analyses			
Data access and cleaning methods		..		RECORD 12.1: Authors should describe the extent to which the investigators had access to the database population used to create the study population.<br><br>RECORD 12.2: Authors should provide information on the data cleaning methods used in the study.	Methods
Linkage		..		RECORD 12.3: State whether the study included person-level, institutional-level, or other data linkage across two or more databases. The methods of linkage and methods of linkage quality evaluation should be provided.	Methods
**Results**					
Participants	13	(a) Report the numbers of		RECORD 13.1: Describe in detail the	Results

		individuals at each stage of the study (*e.g.*, numbers potentially eligible, examined for eligibility, confirmed eligible, included in the study, completing follow-up, and analysed)<br>(b) Give reasons for non-participation at each stage.<br>(c) Consider use of a flow diagram		selection of the persons included in the study (*i.e.*, study population selection) including filtering based on data quality, data availability and linkage. The selection of included persons can be described in the text and/or by means of the study flow diagram.	
Descriptive data	14	(a) Give characteristics of study participants (*e.g.*, demographic, clinical, social) and information on exposures and potential confounders<br>(b) Indicate the number of participants with missing data for each variable of interest<br>(c) *Cohort study* - summarise follow-up time (*e.g.*, average and total amount)			Table 1 Results
Outcome data	15	*Cohort study* - Report numbers of outcome events or summary measures over time<br>*Case-control study* - Report numbers in each exposure category, or summary measures of exposure<br>*Cross-sectional study* - Report numbers of			Figures 1-2

		outcome events or summary measures			
Main results	16	(a) Give unadjusted estimates and, if applicable, confounder-adjusted estimates and their precision (e.g., 95% confidence interval). Make clear which confounders were adjusted for and why they were included<br>(b) Report category boundaries when continuous variables were categorized<br>(c) If relevant, consider translating estimates of relative risk into absolute risk for a meaningful time period	Table 1		Results
Other analyses	17	Report other analyses done—e.g., analyses of subgroups and interactions, and sensitivity analyses	Sensitivity analysis methods and results		Appendix
**Discussion**					
Key results	18	Summarise key results with reference to study objectives	First paragraph of discussion		
Limitations	19	Discuss limitations of the study, taking into account sources of potential bias or imprecision. Discuss both direction and magnitude of any potential bias	Limitations section	RECORD 19.1: Discuss the implications of using data that were not created or collected to answer the specific research question(s). Include discussion of misclassification bias, unmeasured confounding, missing	Limitations section

				data, and changing eligibility over time, as they pertain to the study being reported.	
Interpretation	20	Give a cautious overall interpretation of results considering objectives, limitations, multiplicity of analyses, results from similar studies, and other relevant evidence	Limitations section and last paragraph		Discussion
Generalisability	21	Discuss the generalisability (external validity) of the study results	Last paragraph		Discussion
**Other Information**					
Funding	22	Give the source of funding and the role of the funders for the present study and, if applicable, for the original study on which the present article is based	abstract		Acknowledgment section
Accessibility of protocol, raw data, and programming code		..		RECORD 22.1: Authors should provide information on how to access any supplemental information such as the study protocol, raw data, or programming code.	Methods section

Reference:

1. Benchimol EI, Smeeth L, Guttmann A, Harron K, Moher D, Petersen I, Sørensen HT, von Elm E, Langan SM, the RECORD Working Committee. The REporting of studies Conducted using Observational Routinely-collected health Data (RECORD) Statement. *PLoS Medicine* 2015; in press.
